# The burden of heatwave-related preterm births and associated human capital losses in China

**DOI:** 10.1038/s41467-022-35008-8

**Published:** 2022-12-13

**Authors:** Yali Zhang, Shakoor Hajat, Liang Zhao, Huiqi Chen, Liangliang Cheng, Meng Ren, Kuiying Gu, John S. Ji, Wannian Liang, Cunrui Huang

**Affiliations:** 1 School of Public Health, Sun Yat-sen University, Guangzhou, China; 2 Vanke School of Public Health, Tsinghua University, Beijing, China; 3 Centre on Climate Change and Planetary Health, London School of Hygiene and Tropical Medicine, London, UK; 4 The State Key Laboratory of Numerical Modeling for Atmospheric Sciences and Geophysical Fluid Dynamics, Institute of Atmospheric Physics, Chinese Academy of Sciences, Beijing, China; 5 Institute of Healthy China, Tsinghua University, Beijing, China

**Keywords:** Epidemiology, Environmental impact

## Abstract

Frequent heatwaves under global warming can increase the risk of preterm birth (PTB), which in turn will affect physical health and human potential over the life course. However, what remains unknown is the extent to which anthropogenic climate change has contributed to such burdens. We combine health impact and economic assessment methods to comprehensively evaluate the entire heatwave-related PTB burden in dimensions of health, human capital and economic costs. Here, we show that during 2010-2020, an average of 13,262 (95%CI 6,962-18,802) PTBs occurred annually due to heatwave exposure in China. In simulated scenarios, 25.8% (95%CI 17.1%-34.5%) of heatwave-related PTBs per year on average can be attributed to anthropogenic climate change, which further result in substantial human capital losses, estimated at over $1 billion costs. Our findings will provide additional impetus for introducing more stringent climate mitigation policies and also call for more sufficient adaptations to reduce heatwave detriments to newborn.

## Abstract (web summary)

Frequent heatwaves caused by global warming can lead to an increased risk of preterm birth, however, what remains unknown is the extent to which anthropogenic climate change has contributed to such burdens. Here, the authors combine health impact and economic assessment methods to comprehensively evaluate the entire preterm birth burden of heatwave exposure.

## Introduction

Under continued climate change, children born today will experience a world with significantly increasing and intensifying weather extremes, particularly heatwaves, which deeply affect the health and well-being over their life course^1,2^. Compared to other vulnerable groups, developing fetus and young children are more biologically and psychologically vulnerable to heat stress, suffering immediate and long-term impacts on physical health and human potential^3–5^. Growing evidence shows that heat stress can increase the risk of adverse birth outcomes, in particular preterm birth (PTB)^6^. However, few studies have transformed the exposure-response relationships between heatwaves and PTB into attributable burdens at the regional or national scales^7^. Compared to the relative or attributable risks of PTB from heat stress, heatwave-attributable cases of PTB are more visual for the public and politicians to understand and convenient to make comparison with other climate-related disease burdens.

Considering that both natural and anthropogenic forcings can cause actual climate variabilities, an emerging body of studies are dedicated to detecting the independent contribution of anthropogenic climate change to health effects^8,9^. However, such research to date still focused on heat-related mortality, which mostly occur in older groups rather than newborns or children. What remains unknown is the contribution that anthropogenic climate change is already making to existing PTB burden, even though such knowledge has the potential to better address political inertia on climate actions by demonstrating that serious impacts of anthropogenic climate change are occurring on our children. Assessments of the burden of PTB attributed to heatwaves, especially those induced by anthropogenic forcings, are therefore urgently needed.

Furthermore, substantial evidence from life-course epidemiology and other disciplines suggests that individuals born prematurely are at higher risk of a range of adverse health, cognition, education and social-behavior impacts in the later life^10–12^. Such additional impacts should also be considered and incorporated for a more complete assessment of the true burden of heatwave-related PTB. However, studies assessing the effects of climate change on PTB rarely consider the longer-term consequences of such birth outcome on health and other aspects of human capital, and so our understanding of the true costs of PTB to health and societies remains inadequate. Human capital is an important concept in economic research that comprises multiple dimensions of human potential including health, cognition and non-cognitive abilities, functioning as an important contributor to economic development^13–15^. Compared to traditional health indicators such as disability-adjusted life years (DALY), evaluating human capital losses due to heatwave-related PTB is more conducive to provide a more holistic assessment of long-term impacts of heatwave exposure in early life.

Such research is particularly important for China, a country ranked second in the worldwide PTB burden with more than one million PTBs a year^16^ and endures a heavy disease burden from high temperatures^17^. Hence, this study aims to estimate the number of PTB cases in China over the past decade that are attributed to heatwaves both from actual climate variability, and for the first time, those likely caused by anthropogenic climate change, and in addition, to model the long-term human capital consequences of such PTB impacts. The economic costs of attributable PTB cases and human capital consequences are further estimated for more fully assessment and more complete comparison between the immediate and subsequent impacts of heatwaves.

### Associations between heatwave exposure and PTB in China

Our analyses proceeded in four steps. First, we need to obtain convincing heatwave-PTB associations in China. A recent global meta-analysis published in BMJ has reported a pooled relative risk (RR) of 1.16 (95%CI 1.10-1.23) for PTB associated with the heatwave exposure in the last gestational week or on the day of delivery^6^; however, such evidence is still lacking on a national scale in China. To obtain a nationally representative exposure-response relationship, we used an extended Cox regression to investigate the effect of heatwave exposure during the final gestational week (namely experiencing at least one heatwave) based on a birth cohort of China. This study found the RR for PTB was 1.19 (95%CI 1.09-1.29) due to the heatwave exposure in the last week of gestation (details in Supplementary Methods). We finally used this exposure-response relationship to estimate the heatwave-attributable PTBs in China.

### Spatial-temporal dynamics of heatwave distribution in the past decades

**Fig. 1:**
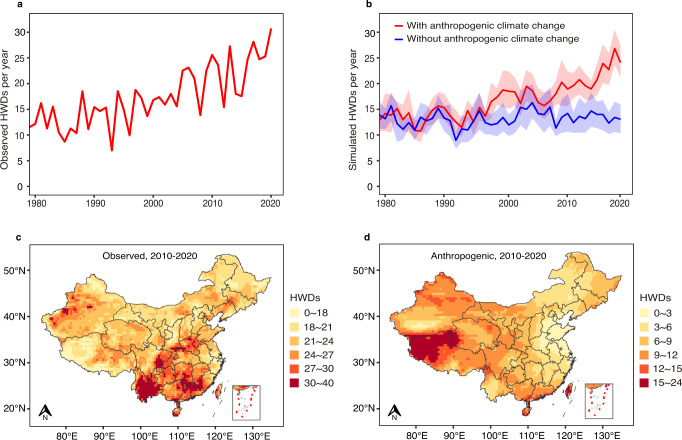
Spatial-temporal distribution of heatwave days in actual climate and those caused by anthropogenic climate change in China. HWDs, heatwave days. **a** The temporal trend of observed national HWDs in actual climate during 1979-2020. **b** Temporal trends of simulated HWDs in the factual (with anthropogenic climate change) and counterfactual (without anthropogenic climate change) scenarios. Solid lines denote the annual mean HWDs across the 10 General Circulation Models and the shaded areas correspond to 95%CIs across the mean estimates. The differences between HWDs of the two scenarios are HWDs attributed to anthropogenic climate change. **c** Spatial distribution (at a 0.5-degree spatial resolution) of annual average HWDs in actual climate during 2010-2020. **d** Spatial distribution of annual average HWDs induced by anthropogenic warming.

In the second step, heatwave that proved to be associated with PTB risk was identified nationally to display the heatwave exposure both in actual climate (based on the observed temperatures) and those induced by anthropogenic climate change (based on two simulated climate scenarios) (Supplementary Methods). In actual climate, heatwave days in China exhibit an upward trend during 1979–2020, with most annual heatwave durations above 20 days occurring since 2010 (Fig. 1a). During 2010–2020, heatwaves were mainly concentrated in southern China, with few in central China (Fig. 1c).

In the factual scenario from climate simulations (to model the actual climate), heatwave days and temporal trends are similar to the observed climate (Fig. 1b), suggesting a good simulation of the modelling to reality. This trend was more stable in the counterfactual scenario (to model the hypothetical climate without anthropogenic forcings) as only natural forcings were considered (Fig. 1b). The differences between heatwave days of the two scenarios are thus interpreted as the additional heatwave days induced by anthropogenic forcings, which were evident since the 1990s and increased significantly during the period of 2015–2020 (Fig. 1b). In contrast to observed heatwaves, heatwaves due to anthropogenic climate change were mainly distributed in the Tibetan Plateau, western and southern China (Fig. 1d).

### PTB burden attributable to heatwaves during 2010–2020

**Fig. 2:**
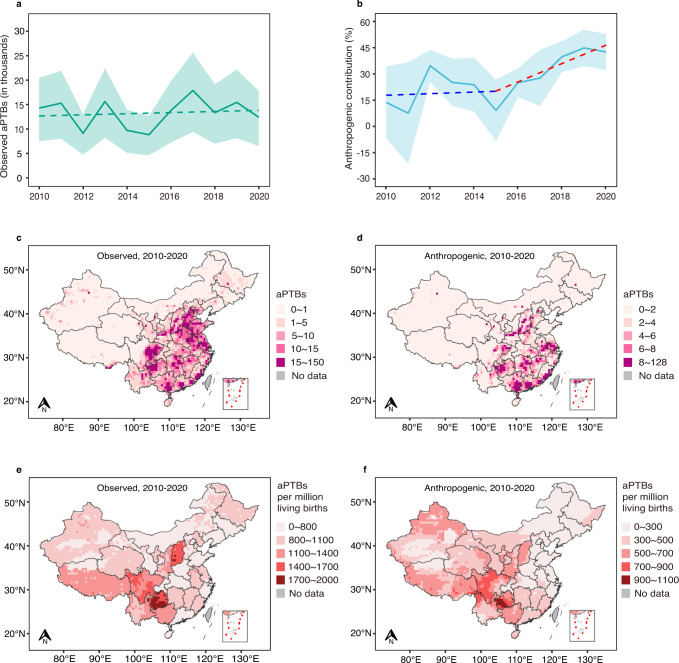
Heatwave-attributable preterm births in actual climate and those induced by anthropogenic climate change in China during 2010-2020. aPTBs, heatwave-attributable preterm births. **a** The temporal trend of aPTBs in actual climate which were based on the observed HWDs during 2010-2020. The solid line shows the point estimates with the shading encompassing their 95%CIs, and the dashed line shows the linear regression. **b** The temporal trend of the proportion of aPTBs induced by anthropogenic warming to aPTBs in the factual scenario. The solid line and shade area correspond to the proportion estimates and their 95%CIs, respectively. The blue dashed line fits the proportions in 2010-2015, while the red dashed line fits the proportions during 2016-2020. **c** Spatial distribution of annual average aPTBs in actual climate in 0.5-degree grids. **d** Spatial distribution of the annual average aPTBs in relation to anthropogenic warming. **e** Spatial distribution of annual average aPTBs per million living births in actual climate. **f** Spatial distribution of aPTBs per million living births that were induced by anthropogenic climate change.

In the third step, we estimated the national heatwave-attributable PTB burden in actual climate (based on observed temperatures) and those induced by anthropogenic climate change (based on simulated data). Estimates were limited to the period 2010–2020 when heatwave days were most numerous, and the impact of anthropogenic climate change was most evident (Fig. 1a, b). Nationally, heatwave-associated PTBs in actual climate amounted to an annual average of 13,262 (95%CI 6,962-18,802) cases, corresponding to 2.6% (95%CI 1.4%-3.7%) of warm-season PTBs annually and 881 (95%CI 461-1,244) attributable PTBs per million living births a year (Supplementary Table 1). Overall, there was a slight increase in heatwave-related PTBs during 2010–2020 (Fig. 2a) and the number of attributable PTBs per million living births were higher in Guizhou, Shanxi and Sichuan (Fig. 2e).

The heatwave-related PTBs induced by anthropogenic climate change gave an average of 4,609 (95%CI 711-6,110) cases annually, amounting to 25.8% (95%CI 17.1%-34.5%) of those in the factual scenario (Supplementary Table 2). This proportion was stable around 20% during 2010–2015, but sharply increased with an average of more than 30% in 2016–2020 (Fig. 2b), which was mainly due to a significant rise of heatwave days associated with the anthropogenic forcings after 2015 (Supplementary Table 3). The heatwave-related PTBs per million living births caused by anthropogenic climate change were mainly concentrated in western and southern China (Fig. 2f).

### Human capital consequences of heatwave-attributable PTBs

In the final step, we estimated human capital consequences of annual attributable PTBs as well as associated economic costs. Owing to the broad concept of human capital and the lack of consistent measurements, we summarized three dimensions (i.e., health, cognition, and non-cognition) of human capital and then selected most common outcomes of PTB for each dimension to comprehensively measure the PTB-related human capital impact (Supplementary Methods). The selected outcomes include neonatal mortality, child asthma, type 1 and 2 diabetes mellitus (health dimension), reduced IQ (cognition), autism spectrum disorder (ASD) and attention deficit hyperactivity disorder (ADHD) (non-cognition). The scientific evidence of their relationships with PTB is shown in Supplementary Table 4.

Based on observational data, we estimate that the average heatwave-related PTBs per year in actual climate (13,262 cases) can lead to 936 cases of additional health outcomes, 1,329 cases of additional non-cognitive problems and more than 111,400 IQ points lost, namely 7.4 reduced IQ points per 1,000 living births. The total economic costs of human capital losses caused by annual attributable PTBs, without considering neonatal mortality, were estimated at nearly $3 billion (in 2015 USD), three times the direct cost of heatwave-related PTBs which was about $1 billion per year (Table 1). By contrast, the human capital losses due to anthropogenic climate change are also significant, whose total economic costs are expected to exceed $1 billion annually (Table 1).

**Table 1:** Human capital losses of annual heatwave-related preterm births in actual climate and those caused by anthropogenic climate change in China

Human capital dimensions	Annual aPTBs and related outcomes	N (Cases or IQ points) (95% CI)	Economic cost per outcome (2015$, in millions)	Cost of aPTBs or entire human capital (2015$, in millions)
**Actual climate (observed)**				
	aPTBs	13262 (6962, 18802)	929.7	929.7
Health	Neonatal death	178 (93, 253)	—	2957.5
	Child asthma	125 (66, 177)	2.9	
	T1D (age<15)	5 (3, 8)	1.0^*^	
	T2D (age>44)	628 (329, 891)	30.5^*^	
Cognition	Reduced IQ	111400 (58480, 157936)	1258.6	
Non-cognition	ASD	876 (460, 1243)	1582.0	
	ADHD	453 (238, 643)	82.5	
**Anthropogenic climate change (simulated)**				
	aPTBs	4609 (711, 6111)	323.1	323.1
Health	Neonatal death	62 (9, 82)	—	1027.0
	Child asthma	43 (7, 58)	1.0	
	T1D (age<15)	2 (0, 3)	0.4^*^	
	T2D (age>44)	218 (34, 290)	10.6^*^	
Cognition	Reduced IQ	38715 (5972, 51332)	437.4	
Non-cognition	ASD	304 (47, 404)	549.0	
	ADHD	157 (24, 209)	28.6	

aPTBs, heatwave-attributable preterm births, reported as the annual average number; CI, confidence interval; T1D, type 1 diabetes; T2D, type 2 diabetes; IQ, intelligence quotient; ASD, autism spectrum disorder; ADHD, attention deficit hyperactivity disorder. *these costs are in 2014 USD.

## Discussion

Taken together, this study provides a holistic assessment of the entire PTB burden attributable to heatwaves, which combines the immediate disease burden of PTB with subsequent human capital losses and associated economic costs. We further provide the first ever estimate of the contribution that anthropogenic climate change is already making to PTB burden. We find that heatwaves have led to significant PTB burden both on health and later-life human capital, a quarter of which can be attributed to anthropogenic climate change that can totally cost over $1 billion economic losses from annual attributable PTBs. By demonstrating the significant contribution of anthropogenic climate change to PTB-related health and human capital burden, our study provides additional compelling reasons to implement more stringent policies to mitigate climate change.

To data, a global burden of PTB attributable to heatwaves remains unknown^17,18^. In this study, we find that heatwave-related PTBs in China are substantial (13,262 cases averagely per year), even comparable to the number of heatwave-related deaths during the same period. According to the 2021 China Report of Lancet Countdown on Health and Climate Change, there were 15,413 deaths on average a year attributable to heatwaves during 2010–2020^18^. We further conclude that a quarter (25.8%) of heatwave-related PTBs on average per year during our study period can be attributed to anthropogenic climate change. This proportion is very significant and close to the contribution that anthropogenic climate change is making to heat-related deaths, which was around 20% in China during 1991–2018^9^. We also observed the proportion has been rising since 2015 and even approached 40% in recent years (Fig. 2b). These findings together underscore the serious and ever-increasing impacts of anthropogenic climate change on the health and wellbeing of the current and future generations.

This study attempts to evaluate the long-term consequences of PTB from the perspective of human capital. Previous studies of health and economic burden tend to quantify DALY^19^ or quality-adjusted life years (QALY)^20^, which are calculated by the lost healthy life years or quality of life years due to premature death and disease, thus still confined to the health aspect. By contrast, our assessment of human capital demonstrates significant consequences of heatwave-related PTBs in the long run, especially for impacts in cognition and non-cognition dimensions which contributed most to the human capital losses and costs. And these cognitive and non-cognitive impairments (i.e., reduced IQ, ASD and ADHD) tend to last for the whole life. Hence, our conceptual framework of human capital would provide a fuller picture of the long-term impacts over the life course, which deserves more attainment when evaluating the full-scale burden of early-life exposure in future research.

Moreover, the monetization analysis in our study indicates that the costs of associated human capital losses in subsequent decades are much more than the direct cost of heatwave-related PTBs. The IPCC Sixth Assessment Report have concluded that the mid- and long-term impacts of climate change on health are multiple times higher than currently observed^21^. We believe our monetization results may extend the evidence that the socio-economic impacts of climate change in the medium to long term can also be magnified through accumulated human capital impact from the immediate damages to the newborns and young children. In other words, the health co-benefits of climate change mitigation could be more substantial if considered the potential benefits of avoidable human capital losses in the long-term, which may greatly offset the economic costs of climate mitigation actions.

Urgent need for simultaneous actions on two fronts has been proposed in recent research, i.e., to protect children today from climate hazards (climate change adaptation) and to attack the root problem by sufficient actions (climate change mitigation)^22^. However, the implementation highly depends upon the capacity and effectiveness of governance and decision-making processes^21^. Our research underscores the necessity of more timely and sufficient adaption support in warm seasons from government to reduce the vulnerability of pregnant mothers, especially those have no access to air conditioning. Reducing emissions of greenhouse gases and strengthening natural carbon sinks is inevitable from a long-term perspective, but this progress is likely to stall in the next decade due to the post-COVID recovery. By demonstrating that human-induced climate change is already having a major impact on PTB-related health and human capital, our findings will provide additional impetus for decision-makers to introduce and implement more stringent climate mitigation policies.

Some limitations of this study should be acknowledged. Firstly, China is a big country with diverse geography and climate, and one exposure-response function of heatwave and PTB may not reflect the situation of whole country. Despite substantial evidence on associations of ambient temperatures with PTB in China^23–25^, however, research on the effect of heatwaves on PTB is still rare. Only a recent study conducted in Guangzhou, China, reported relative risks over 1.37 (95%CI 1.20-1.55) of PTB associated with heatwaves (with similar definition) in the last gestational week^26^. Our estimate (RR = 1.19, 95%CI 1.09-1.29), which was based on 104 thousand newborns from climate-diverse provinces across China, is lower than that in Guangzhou, but very close to the result of the most recent global meta-analysis, which reported a pooled relative risk of 1.16 (95%CI 1.10-1.23) for the short-term effect of heatwave exposure^6^. Hence, the estimate of heatwave-PTB relationship we used (RR = 1.19) is arguably more applicable to date to estimating the national attributable PTBs. Secondly, the associations of PTB with multiple human capital indicators as well as the unit economic costs were derived from studies in different countries, which may not be readily applicable to China and brought uncertainties in assessing human capital losses. We have endeavored to choose results from credible studies in China or meta-analyses worldwide to reduce these uncertainties. Nevertheless, the monetization in this study still provides a general indication that the consequences of early-life heatwave exposure are far-reaching because they can additionally generate substantial societal and economic costs beyond the immediate health impacts.

In conclusion, heatwaves have caused a tremendous disease burden for preterm births in China, of which a quarter can be attributed to anthropogenic climate change. Such PTB effect can further lead to significant losses of human capital and billions of economic costs in the near future. Hence, stringent policies and regulations designed to mitigate climate change are urgently needed, which will bring huge health co-benefits by avoiding early-life health damages and associated human capital losses. More assessments on the early-life impacts of anthropogenic warming are still needed to motivate stronger actions on climate change adaptation and mitigation. The study also provides an innovative analytical framework for a more holistic assessment on other health impacts of climate change.

## Methods

### Observed temperatures and demographic data

We obtained observed daily maximum temperatures from the latest global atmospheric reanalysis version 5 of the European Centre for Medium-Range Weather Forecasts (ERA5) datasets^27^ and annual population numbers from hybrid gridded demographic data^28^. Both temperature and population data were extracted from 1 January 1979 to 31 December 2020 at a 0.5-degree spatial resolution for whole country.

The annual birth rates of 31 provinces of China during 2010–2020 were derived from the Chinese national statistical yearbooks. The proportions of monthly living births were calculated as the fraction of monthly living births in the births of that year, which were derived from the sixth national population census of China (November 1, 2009-October 31, 2010) for they have not been published by the up-to-data seventh national population census (2020). The birth rates and monthly birth proportions were obtained for 31 provinces of China and then matched to each grid inside the province with same values.

### Simulated datasets for the factual and counterfactual climate scenarios

Two climate scenarios have been defined to separate the additional impact of anthropogenic climate change in recent studies^8,9^. One is the factual scenario which accounts for both natural and anthropogenic forcings to simulate the actual historical climate. The other is the counterfactual scenario that approximates a hypothetical climate without anthropogenic forcings. The two scenarios were based on simulation runs from the Detection and Attribution Model Intercomparison Project (DAMIP)^29^. DAMIP is the component of the Coupled Model Intercomparison Project Phase 6 (CMIP6) that aims to assess the individual contributions of anthropogenic and natural forcings on past and future changes in global and regional climate. Several experiments were developed in DAMIP to generate different climate simulations to facilitate analysis of contributions of external forcings^29^. For the factual scenario, we used historical climate simulations from the experiment “CMIP6 historical simulation and SSP2-4.5”, which considered all types of natural and anthropogenic forcings to mimic the actual historical climate. The corresponding counterfactual climate data consists of the simulations of the ‘hist-nat’ experiment, which only considered the natural forcings (solar irradiance and stratospheric aerosols) to approximate a hypothetical climate without human influences.

This study included the simulations of ten General Circulation Models from these two experiments, for which relevant data were available at the time of analysis (1979–2020). Detailed information of models can be seen in Supplementary Table 5. The temperature series for two scenarios can be extracted from the DAMIP climate database. Location-specific series of daily maximum temperature we used were extracted from the globally gridded datasets (https://esgf-node.llnl.gov/search/cmip6/). We extracted the temperature simulations at a 0.5-degree spatial resolution from 1979 to 2020 and used the observed data from ERA5 to bias-correct the simulated temperature series following a method described elsewhere^9,30^.

### Baseline prevalence of PTB in China

PTB was defined as delivery prior to 37 completed weeks of gestation. Owing to the lack of officially published national and provincial PTB prevalence rates in China, we reviewed previous studies on PTB prevalence in China and selected the most credible evidence for analyses. We first used the annual increase rate of national PTB prevalence (1.3%) between 2012 and 2018 reported by the study of Deng et al.^31^, which utilized the data of China’s National Maternal Near Miss Surveillance System (NMNMSS) but did not report the provincial PTB rates. Then, we combined the provincial PTB rates between 2015 and 2016^32^, which were reported by another study based on 89 nationally representative hospitals in China. With the annual increase rate of PTB prevalence (1.3%) and provincial PTB rates in a year, we then estimated the provincial PTB rates in each year during 2010–2020.

### Definition of heatwave with a risk of preterm birth

There is no universally accepted definition of heatwave. Previous studies on heatwave-PTB relationships commonly define a heatwave as two or more days with daily temperatures above a predefined threshold^6^. The mostly used thresholds are the 90^th^ or above percentiles of the temperature distribution, which definitions are usually found to have significant PTB effects^6,33–35^. In order to capture PTB effects of heatwaves and better evaluate the heatwave-related PTB burden, we defined a heatwave event as consecutive two or more days with daily maximum temperatures exceeding the 90^th^ percentile of warm-season temperatures during the study period. In this study, the 90^th^ percentile thresholds were based on the cell temperature distribution to identify the gridded heatwaves.

### Data analysis

#### Quantifying heatwave-attributable PTB burden in the past decade

We quantified the PTB burden attributable to heatwaves both in actual climate and those induced by anthropogenic climate change, respectively. First, we need to calculate the daily baseline PTB number per year of each grid (${{{\mbox{basePTB}}}}_{{{\mbox{g}}},{{\mbox{d}}}}$) with the following two formulas.

1 $${{{\mbox{basePTB}}}}_{{{\mbox{g}}},{{\mbox{m}}}}={{{\mbox{Pop}}}}_{{{\mbox{g}}},{{\mbox{y}}}}\cdot {{{\mbox{BirthRate}}}}_{{{\mbox{g}}},{{\mbox{y}}}}\cdot {{{\mbox{PTBrate}}}}_{{{\mbox{g}}},{{\mbox{y}}}}\cdot {{{\mbox{BirthMon}}}}_{{{\mbox{g}}},{{\mbox{m}}}}$$

2 $${{{{{{\rm{basePTB}}}}}}}_{{{{{{\rm{g}}}}}},{{{{{\rm{d}}}}}}}=\frac{{{{{{{\rm{basePTB}}}}}}}_{{{{{{\rm{g}}}}}},{{{{{\rm{m}}}}}}}}{{{{{{{\rm{Month}}}}}}}_{{{{{{\rm{d}}}}}}}}$$

In formula (1), ${{{\mbox{basePTB}}}}_{{{\mbox{g}}},{{\mbox{m}}}}$ refers to the gridded PTB number of each month in warm seasons (May-October) per year, which can be calculated directly by the yearly gridded population (${{{\mbox{Pop}}}}_{{{\mbox{g}}},{{\mbox{y}}}}$), the yearly birth rate of the grid (${{{\mbox{BirthRate}}}}_{{{\mbox{g}}},{{\mbox{y}}}}$), the yearly PTB rate of the gird (${{{\mbox{PTBrate}}}}_{{{\mbox{g}}},{{\mbox{y}}}}$), and the proportions of monthly living births to that in a year of the grid (${{{\mbox{BirthMon}}}}_{{{\mbox{g}}},{{\mbox{m}}}}$). Here, we assumed that the gridded birth rate, PTB rate, and proportions of monthly living births are equal to those of the province that the grid belongs to. With formula (2), we can obtain the daily baseline PTBs in warm seasons per year for each grid (${{{\mbox{basePTB}}}}_{{{\mbox{g}}},{{\mbox{d}}}}$) by dividing the monthly gridded PTBs (${{{\mbox{basePTB}}}}_{{{\mbox{g}}},{{\mbox{m}}}}$) by the number of days of that month (${{{\mbox{Month}}}}_{{{\mbox{d}}}}$), assuming that the monthly baseline PTBs is evenly distributed on each day.

Second, we identified for each day in warm seasons that whether mothers of preterm births on day *d* in grid *g* (${{{\mbox{basePTB}}}}_{{{\mbox{g}}},{{\mbox{d}}}}$) have experienced heatwaves in their last gestational week, in other words, whether there was at least a heatwave event between day (*d-6*) and day *d*. If mothers prematurely on day *d* once experienced heatwaves in the week, then we calculated attributable fraction (AF) as $\frac{\left({{{{{\rm{RR}}}}}}-1\right)}{{{{{{\rm{RR}}}}}}}$ with the relative risk (RR) we have estimated (Supplementary Methods). The attributable fraction means the proportion of PTBs that can be attributed to heatwaves. If there were no heatwaves between day (*d-6*) and day *d*, then the attributable fraction (AF) for day *d* equals 0. With formula (3), we can obtain the number of heatwave-attributable PTBs on each day for each grid (${{{\mbox{aPTB}}}}_{{{\mbox{g}}},{{\mbox{d}}}}$), which can be further summed to obtain the provincial or national attributable PTBs per year.

3 $${{{{{\rm{AF}}}}}}=\left\{\frac{\left({{{{{\rm{RR}}}}}}-1\right)}{\begin{array}{c}{{{{{\rm{RR}}}}}}\\ 0\end{array}}\right.$$

$${{{\mbox{aPTB}}}}_{{{\mbox{g}}},{{\mbox{d}}}}={{{\mbox{basePTB}}}}_{{{\mbox{g}}},{{\mbox{d}}}}\cdot {{\mbox{AF}}}$$

When calculating the heatwave-related PTBs in actual climate, we identified the heatwave exposure for mothers of PTBs on day *d* in grid *g* (${{{\mbox{basePTB}}}}_{{{\mbox{g}}},{{\mbox{d}}}}$) based on the observed temperature series from ERA5 datasets. With the same procedure, we calculated the attributable PTBs for the factual and counterfactual scenarios respectively based on the heatwaves identified by the bias-corrected temperature simulations. The differences between attributable PTBs of the two scenarios are interpreted as the heatwave-related PTBs caused by anthropogenic climate change.

#### Uncertainty analysis for heatwave-attributable PTBs

The major uncertainty in estimating heatwave-related PTBs in actual climate comes from the heatwave-PTB relationship. The uncertainty in the source of heatwave-PTB association is represented by the variance of model coefficients. We have quantified it by producing 1000 samples of model coefficients using Monte Carlo simulations, assuming that the estimated model coefficients followed a multivariate normal distribution. Then, we derived the 95% CIs of heatwave-related PTBs in actual climate based on the RR distribution derived from coefficient samples, which can incorporate the uncertainty from this relationship.

The uncertainties in estimates of heatwave-related PTBs in simulated scenarios are mainly from the heatwave-PTB relationship as well as the variation of simulated temperatures from different scenario models. We quantified these uncertainties by producing 1000 samples of coefficients through Monte Carlo simulation and then generating results for each scenario model. The 95% CIs of heatwave-related PTBs in two simulated scenarios were derived from the 2.5^th^ and 97.5^th^ percentiles of the empirical distribution across coefficient samples and scenario models. In this way, the CIs account for both the imprecision of the heatwave-PTB relationship and the inherent variability of simulated temperatures across different climate models.

#### Quantifying human capital consequences of attributable PTBs

In order to reflect multi-dimensional consequences of PTB in the long run, we estimated the additional impacts of annual heatwave-associated PTBs on the human capital, which comprises multiple dimensions of human potential over the life course^13,14^. We eventually summarized three dimensions (health, cognition and non-cognition) of human capital and selected the most relevant outcomes in each dimension to measure PTB-related human capital consequences (Supplementary Methods). The human capital indicators including neonatal death, child asthma, type 1 and 2 diabetes (health dimension), reduced IQ points (cognition), and ASD and ADHD (non-cognition). The human capital losses are reported as the additional cases of these outcomes due to heatwave-related PTBs.

Besides reduced IQ, the additional cases for each outcome (neonatal death, child asthma, type 1 and 2 diabetes, ASD and ADHD) were all calculated based on four parameters, i.e. annual average heatwave-related PTB number (${{{\mbox{nPTB}}}}_{{{\mbox{heatwave}}}}$) we have estimated; the relative risk for specific human capital outcome due to PTB (${{{\mbox{RR}}}}_{{{\mbox{outcome}}}}$) derived from existing evidence listed in Supplementary Table 4; the national PTB rate (${{{\mbox{P}}}}_{{{\mbox{ptb}}}}$) reported in previous study^32^; and the baseline prevalence of specific human capital outcome in China (${{{{{\rm{P}}}}}}$_outcome_) from studies listed in Supplementary Table 6. The equations based on these parameters are as the following:

4 $${{{\mbox{RR}}}}_{{{\mbox{outcome}}}}=\frac{{{{\mbox{I}}}}_{{{\mbox{e}}}}}{{{{\mbox{I}}}}_{0}}$$

5 $${{{\mbox{P}}}}_{{{\mbox{outcome}}}}=\frac{{{{\mbox{POP}}}}_{{{\mbox{ptb}}}}\cdot {{{\mbox{I}}}}_{{{\mbox{e}}}}+{{{\mbox{POP}}}}_{{{\mbox{non}}}-{{\mbox{ptb}}}}\cdot {{{\mbox{I}}}}_{0}}{{{{\mbox{POP}}}}_{{{\mbox{all}}}}}\\={{{\mbox{P}}}}_{{{\mbox{ptb}}}}\cdot {{{\mbox{I}}}}_{{{\mbox{e}}}}+(1-{{{\mbox{P}}}}_{{{\mbox{ptb}}}})\cdot {{{\mbox{I}}}}_{0}\\={{{\mbox{P}}}}_{{{\mbox{ptb}}}}\cdot {{{\mbox{I}}}}_{{{\mbox{e}}}}+(1-{{{\mbox{P}}}}_{{{\mbox{ptb}}}})\cdot \frac{{{{\mbox{I}}}}_{{{\mbox{e}}}}}{{{{\mbox{RR}}}}_{{{\mbox{outcome}}}}}$$

6 $${{{\mbox{N}}}}_{{{\mbox{add}}}}={{{\mbox{nPTB}}}}_{{{\mbox{heatwave}}}}\cdot ({{{\mbox{I}}}}_{{{\mbox{e}}}}-{{{\mbox{P}}}}_{{{\mbox{outcome}}}})$$

First, as Eq. (4) shows, the relative risk of the specific human capital outcome due to PTB (${{{\mbox{RR}}}}_{{{\mbox{outcome}}}}$) can be represented by the incidence of this outcome in PTB group (${{{\mbox{I}}}}_{{{\mbox{e}}}}$) and that in non-PTB group (${{{\mbox{I}}}}_{0}$). ${{{\mbox{I}}}}_{{{\mbox{e}}}}$ and ${{{\mbox{I}}}}_{0}$are both unknown and need to be computed. In Eq. (5), ${{{\mbox{POP}}}}_{{{\mbox{ptb}}}}$, ${{{\mbox{POP}}}}_{{{\mbox{non}}}-{{\mbox{ptb}}}}$, and ${{{\mbox{POP}}}}_{{{\mbox{all}}}}$ respectively refer to the number of PTB population, non-PTB population and the entire population. After transformation of the formula, the prevalence of specific human capital outcome in the entire population (${{{\mbox{P}}}}_{{{\mbox{outcome}}}}$) can be finally represented by the national PTB rate (${{{\mbox{P}}}}_{{{\mbox{ptb}}}}$), ${{{\mbox{I}}}}_{{{\mbox{e}}}}$ and ${{{\mbox{RR}}}}_{{{\mbox{outcome}}}}$. Owing to the existing values for ${{{\mbox{P}}}}_{{{\mbox{outcome}}}}$, ${{{\mbox{P}}}}_{{{\mbox{ptb}}}}$ and ${{{\mbox{RR}}}}_{{{\mbox{outcome}}}}$, ${{{\mbox{I}}}}_{{{\mbox{e}}}}$therefore can be computed by the final form of Eq. (5). Finally, according to Eq. (6), we can compute the additional cases due to heatwave-related PTBs for specific human capital outcome (${{{\mbox{N}}}}_{{{\mbox{add}}}}$) by multiplying the annual heatwave-related PTB cases (${{{\mbox{nPTB}}}}_{{{\mbox{heatwave}}}}$) and additional incidence of the outcome in PTB group (${{{\mbox{I}}}}_{{{\mbox{e}}}}-{{{\mbox{P}}}}_{{{\mbox{outcome}}}}$).

Additional reduced IQ points were calculated by multiplying the lost IQ points per PTB children and the annual heatwave-related PTB cases. The heatwave-attributable PTBs and human capital losses were further monetized to make better comparison for the direct and long-term consequences of heatwaves (Supplementary Methods).

All analyses were conducted in R (version 4.1.0).

### Reporting summary

Further information on research design is available in the Nature Portfolio Reporting Summary linked to this article.

## Supplementary information

Supplementary Information

Dataset 1

Dataset 2

Dataset 3

Reporting Summary

Description of Additional Supplementary Files

## Data Availability

ERA5 temperature data are downloadable from the website https://www.ecmwf.int/en/forecasts/datasets/reanalysis-datasets/era5. Hybrid gridded demographic data for China are available at https://zenodo.org/record/3768003. Simulated temperature datasets for the factual and counterfactual climate scenarios can be accessed at https://esgf-node.llnl.gov/search/cmip6/. Data on the annual prevalence of preterm birth, the living birth rates, and monthly birth proportions in China are described in Supplementary Data 1-3 respectively.

## Code availability

Codes to reproduce the statistical analysis are made available from https://github.com/YaLi1024/Heatwave-related_PTBs_assessment.
