# Supplementary material for: The burden of heatwave-related preterm births and associated human capital losses in China
Source: Nat Commun. 2022 Dec 13;13:7565. doi: 10.1038/s41467-022-35008-8 (PMC9747907; doi:10.1038/s41467-022-35008-8)
Supplement: Supplementary file 1 — Supplementary Information [file 41467_2022_35008_MOESM1_ESM.pdf]

Supplementary Information

1. Supplementary Methods

1.1 Examining associations between heatwave exposure and PTB in China

Growing evidence has supported the relationships between heatwave exposure and PTB worldwide. The most recent global meta-analysis published in BMJ has reported a pooled relative risk (RR) of 1.16 (95%CI: 1.10-1.23) for PTB associated with the heatwave exposure in short term[1]. In China, however, such evidence on a national scale is still lacking. Only a recent study conducted in Guangzhou, China, reported RR over 1.37 (95%CI: 1.20-1.55) of PTB associated with heatwaves in the last gestational week[2]. One important reason may be the difficulty in obtaining nationally representative pregnancy and birth data in China. Although China's National Maternal Near Miss Surveillance System (NMNMSS) or medical records containing detailed personal information can be a good source for such study, these data are not publicly available. Here, we obtain a nationally representative heatwave-PTB relationship using a multi-province cohort data in China for further analyses.

Investigating the effect of heatwave exposure on PTB. This analysis was based on a multi-province cohort data of China conducted during 2013-2018 to investigate the effects of heatwave exposure on PTB during the last gestational week. The details of the cohort and matched environmental data have been described in our previous study[3]. Our analysis was limited to warm seasons (May-October) during 2014-2018 to focus on heatwave-related PTB only. A total of 104,151 singleton living births in warm seasons were eventually included, in which 4,305 (4.1%) were PTBs. Although heat exposure throughout the whole pregnancy period may be implicated in PTB risk, but short-term exposure (in the last gestational week) provides the most consistent evidence[1]. Hence, we chose the final gestational week as exposure window to explore heatwave-PTB relationship. The heatwaves were identified by consecutive two or more day with daily temperatures above the 90th quantile of the temperature distribution.

An extended Cox proportional hazard model with multiple covariates was utilized to estimate the hazard ratio (relative risk, RR) for PTB due to heatwave exposure by treating PTB as a time-to-event outcome with term births censored at week 37[2]. Heatwave exposure in the last gestational week was assigned as a binary value (yes/no). Value "*yes*" indicated that the mother has experienced at least a heatwave in the last gestational week. In the model, we controlled the time-independent variables including maternal age, BMI, parity, education level, personal behavior risk factors, season of conception, inspection level and neonatal gender. Time-varying variables were also adjusted for in the model, including average levels of relative humidity, ozone(O_3) and fine particulate matter ($PM_{2.5}$) during the entire pregnancy as well as the mean temperatures in the whole gestation except the last week (as a natural cubic spline with three degrees of freedom). Besides, residency districts of the participants were included to control the random effect as well.

Based on the model controlled for time-independent and time-varying variables as well as residency districts, we found that the relative risk (RR) for PTB was 1.19

(95%CI: 1.09-1.29) due to the heatwave exposure in the last week of gestation, which was further used for heatwave-related PTB estimate.

1.2 Identifying spatial-temporal dynamics of heatwaves in the past decades

Identifying heatwaves in actual climate during 1979-2020. The 90th percentile of daily maximum temperatures in warm seasons during 1986-2005 was computed as the threshold to identify heatwaves over long timescales (1979-2020). Based on ERA5 data, the observed heatwaves in warm seasons (May-October) per year were identified by two or more consecutive days with daily maximum temperatures above the threshold of each grid. The heatwaves were identified for each grid (~50km×50km) based on the cell temperature distribution. The gridded heatwave days were then averaged at the national level to display the temporal trend of national heatwave days. Heatwave days in girds were also averaged by 10 years to illustrate the spatial distribution (in grids) of annual average heatwave days during 2010-2020.

Identifying heatwaves from anthropogenic climate change. The additional heatwave days attributed to anthropogenic climate change were derived from the paired runs of ten General Circulation Models for the factual (accounting for natural and anthropogenic forcings) and counterfactual (accounting for natural forcings only) scenarios. Annual heatwave days (in warm seasons) of the two scenarios during 1979-2020 were identified by simulated temperatures respectively. The heatwaves in the factual scenario are defined as two or more consecutive days with daily maximum temperatures above the threshold, which is the 90th quantile of modelled temperatures for the factual scenario in warm seasons during 1986-2005. Noticeably, the threshold used for the factual scenario was then applied to identify heatwaves in counterfactual scenario. The differences between heatwave days of the two scenarios were computed as additional heatwave days induced by anthropogenic climate change.

1.3 Quantifying the human capital consequences of annual attributable PTBs

Three dimensions of human capital. Owing to the broad concept of human capital and the lack of a consistent measurement, previous studies mainly focused on education outcomes as proxies of human capital, such as years of schooling, academic attainment, enrollment rate and illiteracy rate[4-6] . In recent years, several studies has considered the health and non-cognition dimensions of human capital[7-11], and a few of them considered these dimensions at the same time[9,10]. To better reflect long-term impacts of attributable PTBs on human potential, we eventually summarized three dimensions of human capital, namely health, cognition and non-cognition, according to previous studies[7-9,12].

Health is an essential element of human capital, referring to health status including mortality, morbidity and disability[5]. Cognition encompasses a range of complex neurological and psychological processes[13] , which cannot be completely reflected in a single test. Currently, two broad but complimentary constructs—intelligence quotient (IQ) scores and academic performance—are generally used measures of cognition in research. Non-cognitive skills, which are alternatively called socio-emotional skills, refer to personality, goals, character, motivation and etc. that are highly appreciated in the labor market[14]. Also, there were not constant and

standard measurements for non-cognitive skills. Based on current studies relevant to non-cognitive skills[9-11,15,16], we divide non-cognitive skills into two groups: psychological characteristics (personality traits, self-esteem, motivation, self-confidence) and behavioral tendencies (behavioral problems and social interaction tendency).

Outcomes selected to measure human capital impacts of PTB. We selected the most common long-term outcomes of PTB for each dimension to comprehensively measure the PTB-related human capital impacts. According to previous reviews of the PTB-related outcomes, PTB is a risk of a range of health impairments including premature death in neonates and young children[17], respiratory disease such as child asthma, metabolic disease such as glucose intolerance and diabetes mellitus, and cardiovascular disease such as hypertension[18-20]. Substantial evidence also found a higher risk of those born prematurely in cognitive ability, academic performance[21,22] and behavior problems such as ASD and ADHD[23]. Considering the quality of evidence and the availability of data, we eventually selected the neonatal death, child asthma and diabetes mellitus (type 1&2) for health dimension, reduced IQ for cognition, and ASD and ADHD to measure impacts in non-cognitive dimension.

Monetizing annual attributable PTBs and associated human capital losses. The unit economic costs for PTB and human capital outcomes have been reviewed and available in previous studies. We selected a recent systematic review on per-case monetary estimates for multiple child health outcomes to obtain the costs of PTB, child asthma, ASD, ADHD and loss of an IQ point in 2015 USD[24]. The costs for type 1 and type 2 diabetes are supplemented by another study of assessing the long-term outcomes of PTB induced by air pollution, in which the costs of diabetes were reviewed and converted into 2014 USD[25]. However, we did not find an appropriate estimate of the cost of neonatal mortality for analysis, and so it was not incorporated in monetization. All economic costs per case for PTB or specific human capital outcome and relevant references can be seen in Supplementary Table 7. Finally, the total economic costs of human capital impacts, except the neonatal mortality, are summed to compare with the cost of heatwave-attributable PTBs.

2. Supplementary Results

Supplementary Table 1. The annual number of heatwave-related PTBs in 31 provinces of China under actual climate, 2010-2020

Province	Annual average heatwave-related PTBs (n, 95% CI)	Annual average proportions to warm-season PTBs (%, 95% CI)	Annual average aPTBs per million living births (n, 95% CI)
National total	13262 (6962, 18802)	2.61 (1.36, 3.69)	881 (461, 1244)
Sichuan	1204 (633, 1710)	2.61 (1.36, 3.70)	1392 (729, 1969)
Hunan	811 (427, 1154)	2.46 (1.30, 3.51)	951 (502, 1356)
Hebei	799 (418, 1129)	2.55 (1.34, 3.61)	817 (428, 1156)
Anhui	794 (418, 1129)	2.50 (1.32, 3.56)	1045 (552, 1490)
Guangxi	781 (410, 1107)	2.64 (1.35, 3.65)	1194 (612, 1653)
Guangdong	766 (402, 1085)	2.67 (1.41, 3.81)	605 (320, 864)
Guizhou	736 (387, 1045)	2.65 (1.40, 3.77)	1734 (913, 2467)
Yunnan	709 (372, 1004)	2.46 (1.27, 3.43)	1156 (597, 1615)
Shandong	707 (370, 1000)	2.57 (1.36, 3.68)	846 (449, 1213)
Jiangsu	707 (373, 1006)	2.65 (1.41, 3.80)	597 (317, 858)
Henan	653 (342, 923)	2.76 (1.44, 3.88)	571 (298, 804)
Shanxi	643 (336, 909)	2.94 (1.54, 4.17)	1577 (828, 2235)
Jiangxi	522 (276, 744)	2.52 (1.32, 3.57)	936 (491, 1326)
Shaanxi	464 (244, 658)	2.78 (1.47, 3.97)	1117 (591, 1595)
Fujian	426 (224, 605)	2.55 (1.29, 3.49)	954 (484, 1307)
Hubei	394 (208, 562)	2.56 (1.37, 3.69)	627 (335, 904)
Xinjiang	319 (168, 454)	2.53 (1.33, 3.59)	1021 (536, 1448)
Zhejiang	280 (148, 399)	2.55 (1.34, 3.61)	554 (290, 785)
Gansu	233 (123, 229)	2.35 (1.24, 3.36)	760 (402, 1085)
Inner Mongoria	228 (120, 323)	2.45 (1.28, 3.46)	744 (390, 1053)
Heilongjiang	208 (109, 294)	2.83 (1.48, 4.01)	840 (441, 1189)
Xizang	176 (90, 249)	2.82 (1.48, 3.99)	1215 (635, 1715)
Chongqing	157 (83, 225)	2.61 (1.37, 3.70)	611 (321, 867)
Beijing	124 (65, 176)	2.95 (1.58, 4.26)	567 (303, 819)
Qinghai	84 (44, 120)	2.32 (1.23, 3.33)	920 (488, 1316)
Liaoning	83 (44, 119)	2.58 (1.37, 3.70)	325 (173, 467)
Ningxia	70 (37, 100)	2.27 (1.19, 3.22)	930 (487, 1315)
Jilin	57 (30, 81)	2.66 (1.40, 3.79)	328 (173, 467)
Tianjin	55 (29, 78)	2.72 (1.41, 3.80)	571 (296, 800)
Hainan	42 (22, 61)	2.44 (1.22, 3.30)	706 (353, 955)
Shanghai	16 (9, 24)	2.35 (1.23, 3.33)	585 (306, 827)

PTB, preterm birth. CI, confidence interval. Provinces in this table were ranked by average heatwave-related PTBs.

Supplementary Table 2. The annual number of heatwave-related PTBs induced by anthropogenic climate change in 31 provinces of China, 2010-2020

Province	Average aPTBs in two scenarios (n, 95% CI)		Averaged aPTBs due to anthropogenic climate change (n, 95% CI)	Average rates to factual scenario (%, 95% CI)
	Factual (n, 95% CI)	Counterfactual (n, 95% CI)		
National total	14867 (820, 44058)	10240 (94, 40453)	4609 (711, 6110)	25.8 (17.1, 34.5)
Hainan	51 (0, 173)	23 (0, 137)	27 (0, 36)	52.1 (40.8, 63.4)
Guangdong	879 (31, 2623)	417 (1, 2054)	461 (30, 569)	49.2 (37.2, 61.3)
Qinghai	97 (9, 264)	50 (1, 199)	47 (8, 65)	47.8 (38.8, 56.9)
Gansu	273 (23, 734)	149 (1, 570)	123 (22, 164)	45.0 (37.0, 53.1)
Ningxia	85 (7, 228)	46 (0, 173)	39 (7, 55)	44.3 (32.7, 55.9)
Shaanxi	510 (32, 1438)	288 (3, 1119)	222 (29, 319)	42.4 (33.0, 51.7)
Guangxi	878 (7, 3067)	490 (0, 2626)	387 (7, 441)	40.7 (26.3, 55.1)
Guizhou	353 (29, 2531)	211 (0, 2051)	142 (29, 490)	40.1 (32.1, 48.1)
Xinjiang	805 (29, 957)	462 (1, 560)	342 (28, 397)	40.1 (33.2, 47.0)
Fujian	520 (55, 1377)	315 (3, 1156)	204 (52, 221)	37.0 (24.8, 49.3)
Yunnan	797 (21, 2463)	496 (1, 1895)	300 (20, 568)	34.9 (22.9, 47.0)
Sichuan	1323 (83, 4013)	829 (0, 3435)	493 (83, 578)	34.2 (24.0, 44.4)
Xizang	184 (2, 615)	124 (0, 538)	60 (2, 77)	32.7 (26.2, 39.2)
Hunan	957 (80, 2630)	655 (12, 2232)	301 (68, 398)	31.5 (23.1, 39.9)
Hubei	446 (30, 1267)	302 (4, 1085)	143 (26, 182)	31.2 (20.4, 41.9)
Inner-Mongoria	258 (15, 742)	177 (1, 621)	80 (14, 121)	30.4 (21.4, 39.3)
Shanxi	665 (15, 2016)	451 (6, 1672)	213 (9, 344)	30.3 (18.5, 42.1)
Chongqing	175 (13, 543)	122 (0, 482)	52 (13, 61)	28.5 (20.6, 36.5)
Jiangxi	597 (53, 1634)	420 (3, 1447)	177 (50, 187)	28.2 (16.0, 40.4)
Jilin	63 (3, 190)	45 (0, 156)	17 (3, 34)	28.2 (16.9, 39.5)
Heilongjiang	218 (15, 632)	162 (2, 533)	55(13, 99)	26.5 (15.6, 37.4)
Liaoning	94 (3, 283)	70 (0, 242)	23 (3, 41)	23.5 (12.1, 34.9)
Zhejiang	322 (51, 819)	250 (6, 745)	72 (34, 74)	20.4 (9.6, 31.1)
Beijing	133 (2, 407)	102 (0, 332)	30 (2, 75)	20.0 (7.0, 33.0)
Henan	699 (27, 2068)	563 (5, 2004)	135 (22, 64)	17.2 (0.4, 34.0)
Anhui	907 (60, 2551)	753 (7, 2469)	154 (53, 82)	16.7 (6.2, 27.2)
Hebei	905 (24, 2735)	765 (6, 2561)	140 (18, 174)	12.9 (-1.6, 27.4)
Jiangsu	814 (73, 2347)	734 (17, 2245)	80 (56, 102)	9.9 (2.2, 17.6)
Shanghai	20 (5, 60)	18 (3, 52)	2 (1, 7)	9.3 (-0.9, 19.5)
Shandong	779 (21, 2463)	697(10, 2324)	82 (11, 139)	7.8 (-5.6, 21.3)
Tianjin	60 (2, 188)	54 (1, 177)	6 (1, 11)	7.2 (-8.7, 23.1)

PTB, preterm birth; CI, confidence interval. The average rates were the fraction of aPTBs due to that in the factual scenario. Provinces in this table were ranked by the average rate to factual scenario.

Supplementary Table 3. The changes of anthropogenic warming-related PTBs between 2010-2015 and 2016-2020 in 31 provinces of China and the contribution of driving factors to the changes

Province	Average aPTBs in 2010-2015	Average aPTBs in 2016-2020	Changes of average aPTBs (n)	Contribution of HWDs (n)	Contribution of population (n)	Contribution of birth rate (n)	Contribution of PTB rate (n)
National total	3022	6179	3157	2893	96	-98	265
Guangdong	246	709	463	344	33	57	28
Yunnan	176	417	241	220	6	-2	17
Sichuan	356	594	238	193	-3	20	28
Guangxi	267	500	233	219	6	-14	22
Fujian	115	310	195	168	9	7	12
Jiangxi	85	279	194	190	6	-13	11
Hunan	205	398	193	222	2	-49	18
Hebei	40	226	187	195	4	-19	8
Guizhou	241	428	187	163	-6	11	19
Anhui	75	254	179	179	0	-10	10
Shanxi	143	281	138	139	6	-20	13
Shandong	25	136	111	96	1	9	4
Henan	89	179	90	86	0	-4	8
Jiangsu	39	128	89	85	5	-6	5
Hubei	103	191	88	81	-4	2	9
Zhejiang	39	112	73	61	5	2	4
Inner Mongoria	52	109	57	55	3	-5	5
Shaanxi	186	239	53	29	2	10	12
Beijing	14	49	35	28	5	-1	2
Gansu	105	131	26	30	-2	-8	6
Ningxia	28	52	24	21	2	-1	2
Xizang	49	70	20	16	3	-2	3
Chongqing	44	61	17	16	-2	0	3
Liaoning	15	31	16	14	1	0	1
Tianjin	0	13	12	12	1	-1	0
Hainan	24	30	6	6	1	-2	2
Shanghai	1	4	3	3	0	0	0
Jilin	17	20	3	3	0	-1	1
Qinghai	46	47	1	-1	3	-3	3
Heilongjiang	56	56	0	9	1	-13	3
Xinjiang	142	127	-15	12	9	-43	8

HWDs, heatwave days. Contributions of driving factors were based on the decomposition method developed by Global Burden of Diseases Study (GBD) study[26]. Provinces of this table were ranked by the contribution of HWDs.

Supplementary Table 4. The scientific evidence on three human capital dimensions of PTB-related outcomes

Human capital dimensions	PTB-related outcomes	References	Study design and sample size	Study period	Effect estimate
Health	Neonatal mortality	Xu et al. (2019)[27]	A retrospective study included 13,701 preterm neonates born in 15 hospitals in China.	2013-2014	Mortality of preterm neonates was 1.9%
	Child asthma	Been et al. (2014)[28]	A systematic review and meta-analysis with 30 unique studies involving 1,543,639 children (aged 0.5 to 18 years)	1995-2013	OR: 1.46<br>95% CI: (1.29, 1.65)
	T1D and T2D	Li et al. (2014)[29]	A systematic review and meta-analysis with 18 studies including 2,176,480 participants and 22,073 cases (age < 18) for type 1 diabetes; and 5 studies including 31,478 participants and 1,898 cases (middle-aged) for type 2 diabetes	< 2014	T1D_OR: 1.18<br>95% CI: (1.11, 1.25)<br>T2D_OR: 1.51<br>95% CI: (1.32, 1.72)
Cognition	IQ score	Kerr et al. (2011)[30]	A systematic review and meta-analysis with 27 studies covered 7,044 individuals (3,504 delivered preterm and 3,540 at term)	1980-2009	IQ score was 11.94, 95% CI: (10.47,13.42) points lower among children born preterm
Non-cognition	ASD	Agrawal et al. (2018)[31]	A systematic review and meta-analysis with 18 studies (3,366 preterm infants involved) using ASD diagnostic tools to assess at age 1.5 to 21 years old.	<2017	the prevalence rate of ASD in preterm-born children was 7%, 95% CI: (4%, 9%)
	ADHD	Allotey et al. (2017)[32]	A systematic review and meta-analysis with 74 studies involving 64,061 children	1980-2016	OR: 1.6<br>95% CI: (1.3,1.8)

PTB, preterm birth; T1D, type 1 diabetes; T2D, type 2 diabetes; IQ, intelligence quotient; ASD, autism spectrum disorder; ADHD, attention deficit hyperactivity disorder; CI, confidence interval.

Supplementary Table 5. Information of the selected models for both the factual and counterfactual scenarios

Model	Number	Reference
ACCESS-ESM1-5	r1i1p1f1	Ziehn, T. et al. The Australian Earth System Model: ACCESS-ESM1.5. J. South. Hemisph. Earth Syst. Sci. (2020) doi:10.1071/es19035.
CanESM5	r1i1p1f1	Swart, N. C. et al. The Canadian Earth System Model version 5 (CanESM5.0.3). Geosci. Model Dev. Discuss. (2019) doi:10.5194/gmd-2019-177
BCC-CSM2-MR	r1i1p1f1	Xiao-Ge X I N, Tong-Wen W U, Jie ZHANG F Z, et al. Introduction of BCC models and its participation in CMIP6. Advances in Climate Change Research, 2019, 15(5): 533.
CNRM-CM6-1	r1i1p1f1	Voldoire, A. et al. Evaluation of CMIP6 DECK Experiments With CNRM-CM6-1. J. Adv. 443 Model. Earth Syst. (2019) doi:10.1029/2019MS001683.
FGOALS-g3	r1i1p1f1	Li, L. J. et al. The Flexible Global Ocean–Atmosphere–Land System Model Grid- Point Version 3 (FGOALS-g3): Description and Evaluation. J. Adv. Model. Earth Syst. (2020) doi:10.1029/2019MS002012.
GFDL-ESM4	r1i1p1f1	Dunne, J. P. et al. The GFDL Earth System Model version 4.1 (GFDL- ESM4.1): Overall coupled model description and simulation characteristics. J. Adv. Model. Earth Syst. (2020) doi:10.1029/2019MS002015.
IPSL-CM6A-LR	r1i1p1f1	Boucher, O., Servonnat, J., Albright, A. L., Aumont, O. & Balkanski, Y. Presentation and evaluation of the IPSL-CM6A-LR climate model. J. Adv. Model. Earth Syst. (2020) doi:10.1029/2019MS002010.
MIROC6	r1i1p1f1	Tatebe, H. et al. Description and basic evaluation of simulated mean state, internal variability, and climate sensitivity in MIROC6. Geosci. Model Dev. (2019) doi:10.5194/gmd-12-2727-2019
MRI-ESM2-0	r1i1p1f1	Yukimoto, S. et al. The Meteorological Research Institute Earth system model version 2.0, MRI-ESM2.0: Description and basic evaluation of the physical component. J. Meteorol. Soc. Japan 97, 931–965 (2019).
NorESM2-LM	r1i1p1f1	Seland, Ø. et al. The Norwegian Earth System Model, NorESM2 – Evaluation of theCMIP6 DECK and historical simulations. Geosci. Model Dev. Discuss. (2020) doi:10.5194/gmd-2019-378.

* Each model can conduct two experiments to simulate the factual and counterfactual scenarios respectively, and so it has pair of runs for both scenarios.

Supplementary Table 6. The prevalence of PTB-related human capital outcomes in China

Human capital dimensions	PTB-related outcomes	References	Study design and sample size	Study period	Prevalence
Health	Neonatal mortality	China statistical yearbooks 2010-2020	—	2010-2020	Neonatal mortality was from 8.3‰ in 2010 - 3.4‰ in 2020.
	Child asthma	Li et al. (2020)[33]	A systematic review and meta-analysis with 222 articles conducted in China.	1990-2020	In 2010, the prevalence of asthma in Chinese children aged 0-14 years was 2.12%, 95% CI: (1.83-2.51)
	Type 1 and 2 diabetes	Wang et al. (2017)[34]	A nationally representative cross-sectional survey with 170,287 participants in 2013 in mainland China	2013	The overall prevalence of diabetes was 10.9%, 95% CI: (10.4-11.5) *
Cognition	Reduced IQ points	—	—	—	—
Non-cognition	ASD	Wang et al. (2018)[35]	A comprehensive meta-analysis with 44 studies comprising 2,337,321 subjects in mainland China	2009-2015	the overall prevalence rate for ASD in Chinese (aged from 1.6 to 8 years) was 39.23 per 10,000, 95% CI: (28.44, 50.03 per 10,000)
	ADHD	Liu et al. (2018)[36]	A systematic review and meta-analysis with 67 studies covering 642,266 Chinese children and adolescents were included	1980-2016	The prevalence rate of ADHD in mainland China was 6.5% 95% CI: (5.7–7.3)

PTB, preterm birth; T1D, type 1 diabetes; T2D, type 2 diabetes; IQ, intelligence quotient; ASD, autism spectrum disorder; ADHD, attention deficit hyperactivity disorder; CI, confidence interval. * The prevalence rate of diabetes was unspecific for types. Because approximately 95% of diabetes cases are T2D and approximately 5% are T1D, we therefore calculated the prevalence rates of type 1 with 10.9%×0.05 and type 2diabetes with 10.9%×0.95.

Supplementary Table 7. Unit economic costs of PTB and various human capital outcomes

Study Basis	Selected endpoint	Cost of Illness (COI) Definition	Unit Value (2015 USD) [a]
**Preterm birth (PTB)**			
Institute of Medicine (2007)	Any PTB	medical costs + special education costs + lost productivity costs, 3% discount rate	$70,101
**Asthma**			
Nurmagambetov et al. (2018)	Age 3 onset, no persistence into adulthood	medical costs + absentee costs, 3% discount rate	$23,573
**Type 1 diabetes (T1D)**			
Tao et al. (2010)	Onset from ages 3-45	expected lifetime medical costs and income loss	$199,313 (in 2014 USD)
**Type 2 diabetes (T2D)**			
American Diabetes Association (ADA) (2013)	Type unspecified [b]	medical costs+ reduced productivity costs, 3% discount rate	$48,508 (in 2014 USD)
**Loss of an IQ point**			
Grosse et al. (2002) + Grosse et al. (2007) + Perera et al. (2014)	Best estimate value	lost lifetime earnings, 3% discount rate	$11,298
**Autism spectrum disorder (ASD)**			
Buescher et al. (2014)	ASDs without intellectual disability	productivity costs, 3% discount rate	$1,805,941
**Attention deficit hyperactivity disorder (ADHD)**			
Pelham et al. (2007)	ADHD case persisting from ages 5-17	medical costs + education costs + crime and delinquency costs, 3% discount rate	$182,045

[a], the unit values were extracted from two previous studies[24,25] that reviewed and converted the estimates of studies listed here into the costs in 2015 or 2014 USD. [b], because approximately 95% of diabetes cases are type 2 diabetes and approximately 5% are type 1 diabetes, the cost estimates from the Association (ADA) (2013) were supposed to represent the cost of type 2 diabetes.

Supplementary References

1. Chersich MF, Minh Duc P, Areal A, et al. Associations between high temperatures in pregnancy and risk of preterm birth, low birth weight, and stillbirths: systematic review and meta-analysis. *British Medical Journal* 2020; **371**.

2. Wang Q, Li B, Benmarhnia T, et al. Independent and Combined Effects of Heatwaves and PM2.5 on Preterm Birth in Guangzhou, China: A Survival Analysis. *Environmental Health Perspectives* 2020; **128**(1).

3. Wu K, Hu H, Ren Z, et al. Effects of maternal exposure to fine particulate matter on birth weight in 16 counties across China: a quantile regression analysis. *Environmental Research Letters* 2021; **16**(5): 055014 (12pp).

4. Hu Z, Li T. Too hot to handle: The effects of high temperatures during pregnancy on adult welfare outcomes. *Journal of Environmental Economics and Management* 2019; **94**: 236-53.

5. Lim SS, Updike RL, Kaldjian AS, et al. Measuring human capital: a systematic analysis of 195 countries and territories, 1990-2016. *Lancet* 2018; **392**(10154): 1217-34.

6. Dauda RS. Impact of HIV/aids epidemic on human capital development in West Africa. *Int J Health Plann Manage* 2018; **33**(2): 460-78.

7. Lundberg S. Noncognitive skills as human capital. Education, Skills, and Technical Change: Implications for Future US GDP Growth: University of Chicago Press; 2017: 219-43.

8. Qin X, Wang T, Zhuang C. Intergenerational transfer of human capital and its impact on income mobility: Evidence from China. *China Economic Review* 2016; **38**: 306-21.

9. Wulczyn F, Parolini A, Huhr S. Human capital and child protection: A research framework in the CRC context. *Child abuse & neglect* 2020: 104610.

10. Villa KM. Multidimensional human capital formation in a developing country: Health, cognition and locus of control in the Philippines. *Economics & Human Biology* 2017; **27**: 184-97.

11. Hz A, Xq B, Jz C. Do tiger moms raise superior kids? The impact of parenting style on adolescent human capital formation in China. *China Economic Review* 2020; **63**.

12. Goldin C. Human Capital. *Handbook of Cliometrics* 2016: 55-86.

13. Deary IJ. Intelligence. *Annu Rev Psychol* 2012; **63**: 453-82.

14. Kautz T, Heckman JJ, Diris R, Ter Weel B, Borghans L. Fostering and measuring skills: Improving cognitive and non-cognitive skills to promote lifetime success. 2014.

15. Lundberg S. Non-Cognitive Skills as Human Capital. *Nber Chapters* 2015.

16. Palermo TM, Dowd JB. Childhood obesity and human capital accumulation. *Soc Sci Med* 2012; **75**(11): 1989-98.

17. Liu L, Oza S, Hogan D, et al. Global, regional, and national causes of under-5 mortality in 2000–15: an updated systematic analysis with implications for the Sustainable Development Goals. *The Lancet* 2016; **388**(10063): 3027-35.

18. Raju TNK, Buist AS, Blaisdell CJ, Moxey-Mims M, Saigal S. Adults born preterm: a review of general health and system-specific outcomes. *Acta Paediatr* 2017; **106**(9): 1409-37.

19. Natarajan G, Shankaran S. Short- and Long-Term Outcomes of Moderate and Late Preterm Infants. *Am J Perinatol* 2016; **33**(3): 305-17.

20. Nuyt AM, Lavoie JC, Mohamed I, Paquette K, Luu TM. Adult Consequences of Extremely Preterm Birth: Cardiovascular and Metabolic Diseases Risk Factors, Mechanisms, and Prevention Avenues. *Clin Perinatol* 2017; **44**(2): 315-32.

21. McBryde M, Fitzallen GC, Liley HG, Taylor HG, Bora S. Academic Outcomes of School-Aged Children Born Preterm: A Systematic Review and Meta-analysis. *JAMA Netw Open* 2020; **3**(4): e202027.

22. Twilhaar ES, Wade RM, de Kieviet JF, van Goudoever JB, van Elburg RM, Oosterlaan J. Cognitive Outcomes of Children Born Extremely or Very Preterm Since the 1990s and Associated Risk Factors: A Meta-analysis and Meta-regression. *JAMA Pediatr* 2018; **172**(4): 361-7.

23. Ream MA, Lehwald L. Neurologic Consequences of Preterm Birth. *Curr Neurol Neurosci Rep* 2018; **18**(8): 48.

24. Shea E, Perera F, Mills D. Towards a fuller assessment of the economic benefits of reducing air pollution from fossil fuel combustion: Per-case monetary estimates for children's health outcomes. *Environmental Research* 2020; **182**: 8.

25. Kim JJ, Axelrad DA, Dockins C. Preterm birth and economic benefits of reduced maternal exposure to fine particulate matter. *Environ Res* 2019; **170**: 178-86.

26. Stanaway JD, Afshin A, Gakidou E, et al. Global, regional, and national comparative risk assessment of 84 behavioural, environmental and occupational, and metabolic risks or clusters of risks for 195 countries and territories, 1990–2017: a systematic analysis for the Global Burden of Disease Study 2017. *The Lancet* 2018; **392**(10159): 1923-94.

27. Xu F, Kong X, Duan S, et al. Care Practices, Morbidity and Mortality of Preterm Neonates in China, 2013-2014: a Retrospective study. *Sci Rep* 2019; **9**(1): 19863.

28. Been JV, Lugtenberg MJ, Smets E, et al. Preterm birth and childhood wheezing disorders: a systematic review and meta-analysis. *PLoS Med* 2014; **11**(1): e1001596.

29. Li S, Zhang M, Tian H, Liu Z, Yin X, Xi B. Preterm birth and risk of type 1 and type 2 diabetes: systematic review and meta-analysis. *Obes Rev* 2014; **15**(10): 804-11.

30. Kerr-Wilson CO, Mackay DF, Smith GCS, Pell JP. Meta-analysis of the association between preterm delivery and intelligence. *Journal of Public Health* 2012; **34**(2): 209-16.

31. Agrawal S, Rao SC, Bulsara MK, Patole SK. Prevalence of Autism Spectrum Disorder in Preterm Infants: A Meta-analysis. *Pediatrics* 2018; **142**(3).

32. Allotey J, Zamora J, Cheong-See F, et al. Cognitive, motor, behavioural and academic performances of children born preterm: a meta-analysis and systematic review involving 64 061 children. *BJOG* 2018; **125**(1): 16-25.

33. Li X, Song P, Zhu Y, et al. The disease burden of childhood asthma in China: a systematic review and meta-analysis. *J Glob Health* 2020; **10**(1): 010801.

34. Wang LM, Gao P, Zhang M, et al. Prevalence and Ethnic Pattern of Diabetes and Prediabetes in China in 2013. *Jama-J Am Med Assoc* 2017; **317**(24): 2515-23.

35. Wang F, Lu L, Wang SB, et al. The prevalence of autism spectrum disorders in China: a comprehensive meta-analysis. *Int J Biol Sci* 2018; **14**(7): 717-25.

36. Liu AN, Xu YW, Yan Q, Tong L. The Prevalence of Attention Deficit/Hyperactivity Disorder among Chinese Children and Adolescents. *Scientific Reports* 2018; **8**.
