# Supplementary material for: The burden of heatwave-related preterm births and associated human capital losses in China
Source: Nat Commun. 2022 Dec 13;13:7565. doi: 10.1038/s41467-022-35008-8 (PMC9747907; doi:10.1038/s41467-022-35008-8)
Supplement: Supplementary file 5 — Reporting Summary [file 41467_2022_35008_MOESM5_ESM.pdf]

nature portfolio

Corresponding author(s): Cunrui Huang

Last updated by author(s): Nov 12, 2022

Reporting Summary

Nature Portfolio wishes to improve the reproducibility of the work that we publish. This form provides structure for consistency and transparency in reporting. For further information on Nature Portfolio policies, see our Editorial Policies and the Editorial Policy Checklist.

Statistics

For all statistical analyses, confirm that the following items are present in the figure legend, table legend, main text, or Methods section.

n/a	Confirmed	
☐	☒	The exact sample size (n) for each experimental group/condition, given as a discrete number and unit of measurement
☒	☐	A statement on whether measurements were taken from distinct samples or whether the same sample was measured repeatedly
☒	☐	The statistical test(s) used AND whether they are one- or two-sided<br>*Only common tests should be described solely by name; describe more complex techniques in the Methods section.*
☒	☐	A description of all covariates tested
☐	☒	A description of any assumptions or corrections, such as tests of normality and adjustment for multiple comparisons
☐	☒	A full description of the statistical parameters including central tendency (e.g. means) or other basic estimates (e.g. regression coefficient) AND variation (e.g. standard deviation) or associated estimates of uncertainty (e.g. confidence intervals)
☒	☐	For null hypothesis testing, the test statistic (e.g. F, t, r) with confidence intervals, effect sizes, degrees of freedom and P value noted<br>*Give P values as exact values whenever suitable.*
☒	☐	For Bayesian analysis, information on the choice of priors and Markov chain Monte Carlo settings
☒	☐	For hierarchical and complex designs, identification of the appropriate level for tests and full reporting of outcomes
☒	☐	Estimates of effect sizes (e.g. Cohen's d, Pearson's r), indicating how they were calculated

Our web collection on statistics for biologists contains articles on many of the points above.

Software and code

Policy information about availability of computer code

Data collection	All data preparations, analyses and representations were conducted by R (version 4.1.0)
Data analysis	All data preparations, analyses and representations were conducted by R (version 4.1.0)

For manuscripts utilizing custom algorithms or software that are central to the research but not yet described in published literature, software must be made available to editors and reviewers. We strongly encourage code deposition in a community repository (e.g. GitHub). See the Nature Portfolio guidelines for submitting code & software for further information.

Data

Policy information about availability of data

All manuscripts must include a data availability statement. This statement should provide the following information, where applicable:

- Accession codes, unique identifiers, or web links for publicly available datasets
- A description of any restrictions on data availability
- For clinical datasets or third party data, please ensure that the statement adheres to our policy

ERA5 temperature data are downloadable from the website https://www.ecmwf.int/en/forecasts/datasets/reanalysis-datasets/era5. Hybrid gridded demographic data for China are available at https://zenodo.org/record/3768003. Simulated temperature datasets for the factual and counterfactual climate scenarios can be accessed at https://esgf-node.llnl.gov/search/cmip6/. Data on the annual prevalence of preterm birth, the living birth rates, and monthly birth proportions in China are respectively described in Supplementary Data 1-3.

Human research participants

Policy information about studies involving human research participants and Sex and Gender in Research.

Reporting on sex and gender	The research do not involve the sex and gender.
Population characteristics	The research do not involve human participants.
Recruitment	The research do not involve human participants.
Ethics oversight	This study do not need the ethics oversight.

Note that full information on the approval of the study protocol must also be provided in the manuscript.

Field-specific reporting

Please select the one below that is the best fit for your research. If you are not sure, read the appropriate sections before making your selection.

☐ Life sciences ☐ Behavioural & social sciences ☒ Ecological, evolutionary & environmental sciences

For a reference copy of the document with all sections, see nature.com/documents/nr-reporting-summary-flat.pdf

Ecological, evolutionary & environmental sciences study design

All studies must disclose on these points even when the disclosure is negative.

Study description	Based on the observed and simulated temperature series and demographic data, we combined the health impact and economic assessment methods, to estimate the heatwave-related preterm birth (PTB) cases in China over the past decade, and for the first time, to quantify the contribution that anthropogenic climate change is already making to PTB burden. In addition, we further modeled the long-term human capital consequences of such PTB impacts and evaluated associated economic costs for more fully assessment.
Research sample	We used the population numbers in 0.5-degree grids of the whole country from hybrid gridded demographic dataset, combining with the PTB prevalence, birth rates and monthly birth proportions of each province from previous research, national statistical yearbooks and population census respectively, to estimate the national PTB cases per year at a 0.5-degree spatial resolution, which were further used for assessment of the national heatwave-related PTB cases.
Sampling strategy	Owing to the limited evidence of PTB prevalence, we restricted the study period to recent decade, 2010-2020, and extracted the temperature and demographic data for each year from 2010 to 2020 . Since we need to evaluate the national heatwave-related PTB burden, the data of the whole country (at the gridded or provincial level) in the study period were included for analysis. For accuracy of the estimate, these data were all obtained from datasets that are official and authoritative nationally or worldwide.
Data collection	We extracted observed temperature data for China at a 0.5-degree resolution from the latest global atmospheric reanalysis version 5 of the European Centre for Medium-Range Weather Forecasts (ERA5) datasets (https://www.ecmwf.int/en/ forecasts/datasets/ reanalysis-datasets/era5). The simulated temperature data of ten General Circulation Models were collected from datasets of the Coupled Model Inter-comparison Project phase 6 (https://esgf-node.llnl.gov/search/cmip6/). Gridded population numbers of China were obtained from hybrid gridded demographic datasets (https://zenodo.org/record/3768003). Data on the prevalence of PTB, the annual birth rates, and monthly birth proportions of 31 provinces in China during 2010-2020 were extracted from previous study, national statistical yearbooks and population census, respectively. These data were collected by Y.Z., L.Z., H.C. and L.C. .
Timing and spatial scale	We used daily temperature series (both observed and simulated) and population numbers from 2010 to 2020 at a 0.5-degree spatial resolution for the whole country. The PTB prevalence, birth rates and monthly birth proportions from 2010 to 2020 were extracted for each province and matched to the 0.5-degree grids inside the province. All the data were analyzed at the gird level and the results were aggregated to the provincial or national level.
Data exclusions	No data were excluded from the analysis.
Reproducibility	All attempts to repeat the analysis were successful.
Randomization	This study did not involve the randomization because it is a modelling study that quantifies the burden of heatwave-related PTBs based on existing findings and public datasets.
Blinding	Blinding was not applicable in our study as it is a modelling study on assessment of heatwave-related PTB burden.

Did the study involve field work? ☐ Yes ☒ No

Reporting for specific materials, systems and methods

We require information from authors about some types of materials, experimental systems and methods used in many studies. Here, indicate whether each material, system or method listed is relevant to your study. If you are not sure if a list item applies to your research, read the appropriate section before selecting a response.

Materials & experimental systems

n/a	Involved in the study	
☒	☐	Antibodies
☒	☐	Eukaryotic cell lines
☒	☐	Palaeontology and archaeology
☒	☐	Animals and other organisms
☒	☐	Clinical data
☒	☐	Dual use research of concern

Methods

n/a	Involved in the study	
☒	☐	ChIP-seq
☒	☐	Flow cytometry
☒	☐	MRI-based neuroimaging
