# Supplementary material for: The burden of heatwave-related preterm births and associated human capital losses in China
Source: Nat Commun. 2022 Dec 13;13:7565. doi: 10.1038/s41467-022-35008-8 (PMC9747907; doi:10.1038/s41467-022-35008-8)
Supplement: Supplementary file 6 — Description of Additional Supplementary Files [file 41467_2022_35008_MOESM6_ESM.pdf]

Description of Additional Supplementary Files

File Name: Supplementary Data 1
Description: The annual prevalence of preterm birth of 31 provinces in China.

File Name: Supplementary Data 2
Description: The annual living birth rates (per thousand) of 31 provinces in China.

File Name: Supplementary Data 3
Description: The proportions of monthly living births (in warm seasons) to that in a year for 31 provinces in China.
